# Insights on *Mycobacterium leprae* Efflux Pumps and Their Implications in Drug Resistance and Virulence

**DOI:** 10.3389/fmicb.2018.03072

**Published:** 2018-12-13

**Authors:** Diana Machado, Emmanuel Lecorche, Faiza Mougari, Emmanuelle Cambau, Miguel Viveiros

**Affiliations:** ^1^Global Health and Tropical Medicine, Instituto de Higiene e Medicina Tropical, Universidade Nova de Lisboa, Lisbon, Portugal; ^2^Study Group for Mycobacterial Infections (ESGMYC), European Society for Clinical Microbiology and Infectious Diseases (ESCMID), Basel, Switzerland; ^3^Université Paris Diderot, INSERM IAME UMR1137, Sorbonne Paris Cité, Paris, France; ^4^APHP, Groupe Hospitalier Lariboisière Fernand-Widal, Laboratoire de Bacteriologie, Paris, France; ^5^Centre National de Référence des Mycobactéries et Résistance des Mycobactéries aux Antituberculeux, Paris, France

**Keywords:** antimicrobial resistance, efflux pumps, leprosy, mycobacteria, tuberculosis, virulence

## Abstract

Drug resistance in *Mycobacterium leprae* is assumed to be due to genetic alterations in the drug targets and reduced cell wall permeability. However, as observed in *Mycobacterium tuberculosis*, drug resistance may also result from the overactivity of efflux systems, which is mostly unexplored. In this perspective, we discuss known efflux pumps involved in *M. tuberculosis* drug resistance and virulence and investigate similar regions in the genome of *M. leprae*. *In silico* analysis reveals that the major *M. tuberculosis* efflux pumps known to be associated with drug resistance and virulence have been retained during the reductive evolutionary process that *M. leprae* underwent, e.g., RND superfamily, the ABC transporter BacA, and the MFS P55. However, some are absent (DinF, MATE) while others are derepressed (Mmr, SMR) in *M. leprae* reflecting the specific environment where *M. leprae* may live. The occurrence of several multidrug resistance efflux transporters shared between *M. leprae* and *M. tuberculosis* reveals potential implications in drug resistance and virulence. The conservation of the described efflux systems in *M. leprae* upon genome reduction indicates that these systems are potentially required for its intracellular survival and lifestyle. They potentially are involved in *M. leprae* drug resistance, which could hamper leprosy treatment success. Studying *M. leprae* efflux pumps as new drug targets is useful for future leprosy therapeutics, enhancing the global efforts to eradicate endemic leprosy, and prevent the emergence of drug resistance in afflicted countries.

## Introduction

Leprosy and tuberculosis are public health threatening infectious diseases with similar problems of ongoing human-to-human transmission, inherent drug resistance to several antimicrobial agents, propensity to develop resistance to antimycobacterial drugs, and virulence (Singh et al., 2016; Dheda et al., 2017). Whilst there is extensive knowledge about the mechanisms of *M. tuberculosis* drug resistance, less is known about the mechanisms by which *M. leprae* develops drug resistance. *M. leprae* is an obligate intracellular pathogen and one of the few known microorganisms that still cannot be cultured *in vitro* which have been hindering the study of the mechanisms of drug resistance by biochemical and functional studies. The overexpression of multidrug (MDR) efflux pump genes is a common mechanism of antimicrobial resistance in *M. tuberculosis* (da Silva et al., 2016; Machado et al., 2017). Likewise, efflux pumps certainly contribute to drug resistance in *M. leprae*, which is mostly unexplored.

Efflux pumps are one of the most widespread resistance determinants in bacteria. Usually, they are chromosomally encoded and are greatly conserved at both gene and protein level across bacterial species. More than 50 putative efflux pumps have been associated with the transport of several drugs in *M. tuberculosis* (De Rossi et al., 2006; Louw et al., 2009; Kapopoulou et al., 2011; Black et al., 2014; da Silva et al., 2016). Although they are mostly known due to their role in the efflux of antimicrobials, efflux pumps are mainly involved in physiological processes such as cell-to-cell communication, bacterial virulence, cellular homeostasis, detoxification of intracellular metabolites, and intracellular signal trafficking (De Rossi et al., 2006; Martinez et al., 2009; Viveiros et al., 2012; Black et al., 2014; da Silva et al., 2016; Li et al., 2016; Sandhu and Akhter, 2018). Recently, it was shown that the loss of the efflux pump AcrAB in *Salmonella enterica* serovar Typhymurium reduces virulence leading to the accumulation of noxious molecules inside the bacteria reducing the bacterial factors required for infection (Wang-Kan et al., 2017). From a biological point of view, drug resistance and virulence are required for pathogen survival. In normal conditions, the expression of these systems is tightly downregulated by specific transcriptional regulators (Grkovic et al., 2001) and their overexpression is achieved only in the presence of specific stressors capable of binding to the transcriptional regulators. The induction of efflux systems in the presence of inducers such as antimicrobials or host factors during infection promote a low-level resistance phenotype that allows the bacteria to survive during prolonged periods in the presence of drugs contributing for the development and stabilization of resistant phenotypes (Machado et al., 2012; Schmalstieg et al., 2012).

In this perspective, we compared *in silico M. tuberculosis* efflux pumps involved in drug resistance and virulence with those of *M. leprae* investigating their possible involvement in antimicrobial resistance and virulence in *M. leprae*.

## Reductive Evolution

The genome of the non-pathogenic *Mycobacterium smegmatis* mc^2^155 has 7 Mb in size; the genomes of the pathogenic *M. tuberculosis* and *M. leprae* are much smaller in length and for *M. leprae* this is even more dramatic with almost 2000 genes lost in comparison with *M. tuberculosis*. Compared to non-pathogenic mycobacteria, *M. tuberculosis* and *M. leprae* evolved by extensive reductive evolution suggesting that pathogenic mycobacteria evolved toward pathogenicity by the loss of genetic material as the result of niche adaptation. Contrary to *M. tuberculosis, M. leprae* is an obligate intracellular pathogen. Adaptation to a permanent pathogenic lifestyle in constant association with the host led to gene loss toward a minimal gene set, such as those coding for metabolism and respiration, needed for a successful obligate intracellular parasitism (Moran, 2002; Scollard et al., 2006) and limited capacity to survive extracellularly (Cole et al., 2001; Eiglmeier et al., 2001). Of interest is the fact that the G+C content is lower in *M. leprae* pseudogenes (56.5%) than in its active ORFs (60.1%) (Cole et al., 2001). Changes in G+C content during the path of evolution may confer an advantage in response to environmental changes (Mann and Chen, 2010). Free-living organisms have an average G+C content higher than obligatory pathogens and symbionts. The shift toward lower G+C contents and smaller genomes in obligate pathogenic mycobacteria seems to occur in response to environmental adaptation where they encounter low selective pressure (Mann and Chen, 2010). In this context some genes became inactivated, as they are not required in these highly specialized niches meeting the theoretical principles of Morris, Lenski, and Zinser's Black Queen Hypothesis for the symbiotic reductive genome evolution of microorganisms (Morris et al., 2012), now applied to a bacterium and his long-lasting and almost exclusive host—the human being. In this case, *M. leprae* relies on his host functions to live efficiently, losing burdensome genes for functions it does not have to perform for itself (Morris et al., 2012). Nevertheless, it is not clear why *M. leprae* maintains such high number of pseudogenes in the genome. It has been hypothesized that the maintenance of pseudogenes is due to the slow-growth rate (McLeod et al., 2004) or lack of recombination (Bolotin and Hershberg, 2015). Additionally, pseudogene maintenance may allow the bacteria to revert back and forward from a non-functional protein to a functional one (Bolotin and Hershberg, 2015). If true, this may explain why some genes are loss and others are maintained as pseudogenes in *M. leprae* genome.

In this evolutionary context, where an obligate intracellular pathogen evolved to become dependent on his host, the central question of this work is the impact of *M. leprae* genome downsizing on antimicrobial resistance. This can be viewed as the time when the host decides that he no longer wants to maintain this intimate relation and starts antibiotic treatment with the assistance of his clinician and the health system.

## Drug Resistance and Efflux Systems in *M. leprae*

**Figure 1:**
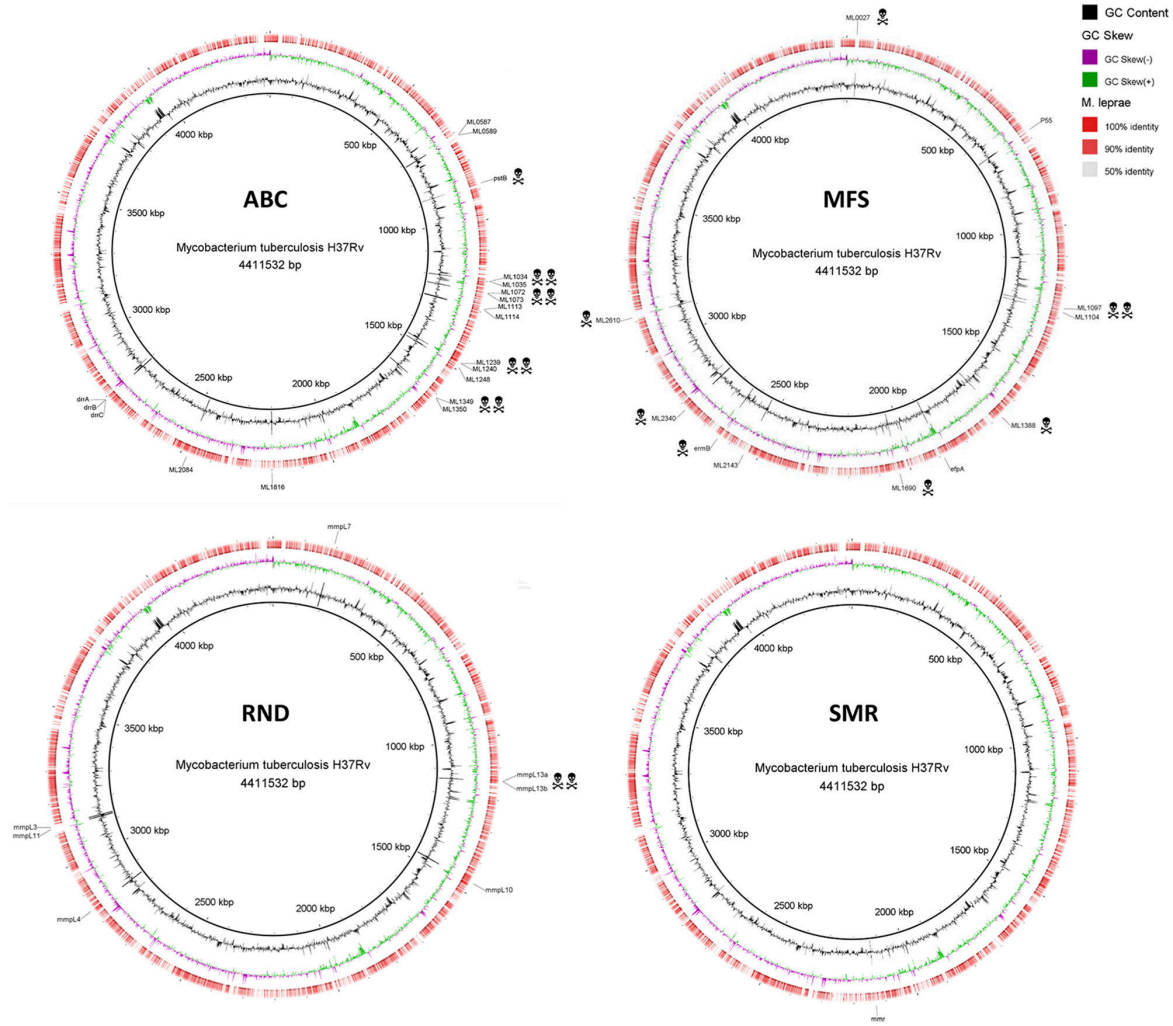
Alignment of *M. leprae* TN predicted CDS regions of efflux transporters to those of *M. tuberculosis* H37Rv. Efflux transporters identified in *M. tuberculosis* are highlighted outside the ring in black. The death heads denote pseudogenes. See Table 1 for locus tag. The circular genomic comparison was generated using BRIG software (Alikhan et al., 2011). CDS, coding DNA sequence.

Several mycobacterial drug efflux pumps have been described in *M. tuberculosis* (Table 1). Comparative analysis of *M. leprae* genome shows the presence of approximately half of these transporters while several others are inactivated or absent, probably lost as consequence of reductive evolution. *In silico, M. tuberculosis* H37Rv genome encodes 267 putative transporters, of which 129 belong to the ATP-binding cassette (ABC) superfamily, 31 to the major facilitator superfamily (MFS), 14 to the resistance nodulation and cell division (RND) superfamily, 1 to the small multidrug resistance (SMR) family, and 1 to the multidrug and toxic compound extrusion (MATE) family. *M. leprae* genome encodes for 114 transporters, of which 62 corresponds to ABC transporters, 6 MFS, 5 RND, and 1 SMR (Elbourne et al., 2017). Those that have been associated with drug resistance in *M. tuberculosis* are discussed below. Alignment visualization of the *M. tuberculosis* and the *M. leprae* whole genome sequences, with the predicted CDS regions of the efflux transporters of *M. leprae* highlighted is shown in Figure 1.

**Table 1:** Putative drug membrane transporters encoded by *M. tuberculosis* H37Rv and its orthologous in *M. leprae* TN.

**Efflux pump family**	**Gene**	Gene locus tag^*^		Identity (%)^**^	***M. tuberculosis***	
		***M. tuberculosis***	***M. leprae***		**Antimicrobial substrates**	**Main references**
**ABC**						
“One gene”	*bacA*	Rv1819c	ML2084	75	RIF, INH, BL, CHL, TET, VAN, MAC, NOV, AGs, AP	Danilchanka et al., 2008; Domenech et al., 2009; Gupta et al., 2010a; Kapopoulou et al., 2011; Li et al., 2015
	*Rv0194*	Rv0194	Absent	-	BL, CHL, STR, TET, VAN, MAC, NOV, EMB, EtBr	Danilchanka et al., 2008; Kapopoulou et al., 2011; Garima et al., 2015
	*pstB*	Rv0933	ML0741c	-	FQs, INH, RIF, EMB	Banerjee et al., 1996, 2000; Braibant et al., 1996; Gupta et al., 2006; Srivastava et al., 2010; Kapopoulou et al., 2011; Brandis and Hughes, 2013; Lu et al., 2014
	*Rv1473*	Rv1473	ML1816c	88	MAC	Kapopoulou et al., 2011
	*Rv2477c*	Rv2477c	ML1248	92	MAC, FQs	Gupta et al., 2010a; Kapopoulou et al., 2011
“Two-genes”	*Rv1218c- Rv1217c*	Rv1218c- Rv1217c	ML1073c- ML1072c	-	BL, NOV, BP, PD, PR, BSP, PA, INH, RIF	Balganesh et al., 2010; Kapopoulou et al., 2011; Dinesh et al., 2013; Wang et al., 2013
	*Rv1273c-*	Rv1273c-	ML1114c-	78	Unknown	Kapopoulou et al., 2011
	*Rv1272c*	Rv1272c	ML1113c	75		
	*Rv1668c- Rv1667c*	Rv1668c- Rv1667c	ML1240c- ML1239c	-	MAC	Kapopoulou et al., 2011
	*Rv1687c- Rv1686c*	Rv1687c- Rv1686c	ML1350c- ML1349c	-	MAC	Kapopoulou et al., 2011
“Three-genes”	*Rv1458c-*	Rv1458c-	ML0590c-	88	RIF, INH, STR, EMB	Hao et al., 2011; Kapopoulou et al., 2011; Caleffi-Ferracioli et al., 2016
	*Rv1457c-*	Rv1457c-	ML0589c-	83		
	*Rv1456c*	Rv1456c	ML0587c	83		
	*Rv2688c-*	Rv2688c-	Absent	-	FQs	Pasca et al., 2004; Gupta et al., 2010a; Kapopoulou et al., 2011
	*Rv2687c-*	Rv1687c-	ML1035	-		
	*Rv2686c*	Rv1686c	ML1034	-		
	*drrA-*	Rv2936-	ML2352c-	85	TET, EMB, MAC, AGs, CHL, RIF, EtBr, NOR, PUR, BCECF, DAU, DOX	Choudhuri et al., 2002; Kapopoulou et al., 2011; Pang et al., 2013; Li et al., 2015
	*drrB-*	Rv2937-	ML2351c-	64		
	*drrC*	Rv2938	ML2350c	79		
**MFS**						
	*Rv0037c*	Rv0037c	ML0027c	-	Unknown	Kapopoulou et al., 2011
	*Rv0191*	Rv0191	ML2610	-	RIF	Kapopoulou et al., 2011; Li et al., 2015
	*emrB*	Rv0783c	ML2224	-	Multiple drugs	De Rossi et al., 2002; Gupta et al., 2010a; Kapopoulou et al., 2011; Brandis and Hughes, 2013; Li et al., 2015
	*Rv0842*	Rv0842	Absent	-	RIF	Kapopoulou et al., 2011; Li et al., 2015
	*Rv0849*	Rv0849	Absent	-	BL, INH, RIF	Kapopoulou et al., 2011; Balganesh et al., 2012
	*Rv0876c*	Rv0876c	ML2143	81	Unknown	Kapopoulou et al., 2011
	*Rv1250*	Rv1250	ML1097	-	INH	Kapopoulou et al., 2011; Garima et al., 2015; Li et al., 2015
	*Rv1258c*	Rv1258c	ML1104c	-	TET, FQs, RIF, CFZ, INH, EMB, ERY, EtBr, SPE	Ainsa et al., 1998; Siddiqi et al., 2004; Gupta et al., 2006; Ramón-García et al., 2006, 2012; Jiang et al., 2008; Kapopoulou et al., 2011; Balganesh et al., 2012; Machado et al., 2012, 2017
	*p55*	Rv1410c	ML0556c	82	TET, AGs, RIF, INH, CFZ	da Silva et al., 2001; Jiang et al., 2008; Ramón-García et al., 2009; Bianco et al., 2011a,b; Kapopoulou et al., 2011; Machado et al., 2012, 2017; Li et al., 2015
	*Rv1634*	Rv1634	ML1388	-	FQs; SKI	De Rossi et al., 2002; Kapopoulou et al., 2011; Harris et al., 2014
	*Rv1672c*	Rv1672c	Absent	-	Unknown	Kapopoulou et al., 2011
	*Rv1877*	Rv1877	Absent	-	RIF, EtBr, ACR, ERY, KAN, TET	De Rossi et al., 2002; Li et al., 2004; Kapopoulou et al., 2011; Louw et al., 2011
	*Rv2265*	Rv2265	Absent	-	Unknown	Kapopoulou et al., 2011
	*stp*	Rv2333c	Absent	-	SPE, TET, RIF	Ramón-García et al., 2007; Kapopoulou et al., 2011; Li et al., 2015
	*Rv2456c*	Rv2456c	Absent	-	Unknown	Kapopoulou et al., 2011
	*Rv2459*	Rv2459	Absent	-	INH, EMB, RIF, EtBr	De Rossi et al., 2002; Gupta et al., 2010b; Kapopoulou et al., 2011; Machado et al., 2012; Li et al., 2015
	*efpA*	Rv2846c	ML1562c	81	INH, RIF, EtBr, ACR, ERY, FQs	Doran et al., 1997; Wilson et al., 1999; Li et al., 2004, 2015; Gupta et al., 2010a; Kapopoulou et al., 2011; Machado et al., 2012, 2017
	*Rv2994*	Rv2994	ML1690	-	STR, RIF	Gupta et al., 2010a; Kapopoulou et al., 2011; Louw et al., 2011
	*Rv3239c*	Rv3239c	Absent	-	Unknown	Kapopoulou et al., 2011
	*Rv3728*	Rv3728	ML2340	-	RIF	Gupta et al., 2010a; Kapopoulou et al., 2011
**RND**						
	*mmpS1-mmpL1*	Rv0403c- Rv0402c	Absent	-	Unknown	Kapopoulou et al., 2011
	*mmpS2-mmpL2*	Rv0506- Rv0507	Absent	-	Unknown	Kapopoulou et al., 2011
	*mmpL3*	Rv0206c	ML2620c	76	SQ109, BM212, AU, IA	Kapopoulou et al., 2011; La Rosa et al., 2012; Tahlan et al., 2012; Li et al., 2014
	*mmpS4-*	Rv0451c-	ML2377	75	CMB, MB, RIF	Kapopoulou et al., 2011; de Knegt et al., 2013; Wells et al., 2013
	*mmpL4*	Rv0450c	ML2378	79		
	*mmpS5-mmpL5*	Rv0677c- Rv0676c	Absent	-	AZ, BDQ, CFZ, TET	Milano et al., 2009; Kapopoulou et al., 2011; Hartkoorn et al., 2014
	*mmpL6*	Rv1557	Absent	-	Unknown	Kapopoulou et al., 2011
	*mmpL7*	Rv2942	ML0137c	69	INH	Choudhuri et al., 1999; Domenech et al., 2005; Kapopoulou et al., 2011; Machado et al., 2012 Pasca et al., 2005
	*mmpL8*	Rv3823c	Absent	-	SQ109	Domenech et al., 2004; Kapopoulou et al., 2011; Li et al., 2014
	*mmpL9*	Rv2339	Absent	-	SQ109	Kapopoulou et al., 2011; Li et al., 2014
	*mmpL10*	Rv1183	ML1231	71	Unknown	Kapopoulou et al., 2011
	*mmpL11*	Rv0202c	ML2617c	73	Unknown	Kapopoulou et al., 2011
	*mmpL12*	Rv1522c	Absent	-	Unknown	Kapopoulou et al., 2011
	*mmpL13a*	Rv1145	ML0971	-	Unknown	Kapopoulou et al., 2011
	*mmpL13b*	Rv1146	ML0972	-	Unknown	Kapopoulou et al., 2011
	*mmpS3*	Rv2198c	ML0877	68	-	Kapopoulou et al., 2011
**SMR**						
	*mmr*	Rv3065	ML1756	79	ACR, EtBr, INH, MAC, FQs, TPP, PY	De Rossi et al., 1998; Kapopoulou et al., 2011; Balganesh et al., 2012; Machado et al., 2012; Rodrigues et al., 2013
**MATE**						
	*dinF*	Rv2836c	Absent	-	AGs, Phleo, sulpha drugs, CPC	Kapopoulou et al., 2011; Mishra and Daniels, 2013

* CDS, coding DNA sequence;

** *determined at protein level; pseudogenes are underlined. ABC, ATP-binding cassette; ACR, acriflavine; AGs, aminoglycosides; AP, antimicrobial peptides; AU, adamantyl ureas; AZ, azoles; BCECF, 2',7'-bis-(2-carboxyethyl)-5(6)-carboxyfluorescein; BDQ, bedaquiline; BL, β-lactams; BP, biarylpiperazines; BSP, bisanilinopyrimidines; CFZ, clofazimine; CHL, chloramphenicol; CMB, carboxymycobactins; CPC, cetylpyridinium chloride; DAU, daunorubicin; DOX, doxorubicin; EMB, ethambutol; ERY, erythromycin; EtBr, ethidium bromide; FQs, fluoroquinolones; IA, indoleamides; INH, isoniazid; KAN, kanamycin; MAC, macrolides; MATE, multidrug and toxic compound extrusion; MB, mycobactins; MFS, major facilitator superfamily; NOR, norfloxacin; NOV, novobiocin; PA, pyrazolones; PD, pyridines; Phleo, phleomycin; PR, pyrroles; PUR, puromycin; PY, pyronin Y; RIF, rifampicin; RND, resistance nodulation division; SKI, imidazoline SKI-356313; SMR, small multidrug resistance; SPE, spectinomycin; STR, streptomycin; TET, tetracycline; TPP, tetraphenylphosphonium; VAN, vancomycin*.

### ABC Transporters

ABC transporters can be divided in those encoded by “one,” “two,” and “three genes” (Table 1). Among those coded by “one gene” in *M. leprae* is ML2084, homologue of BacA that is involved in virulence of *M. tuberculosis* (Domenech et al., 2009) and in the active transport of drugs across the membrane (Table 1). Absent from *M. leprae* genome is the transporter Rv0194, which was the first to be associated with β-lactam transport in *M. tuberculosis*-important taking into account since β-lactams can be useful in *M. leprae* chemotherapy (Danilchanka et al., 2008; Garima et al., 2015). The phosphate-specific ABC transporter (PstB) is known to be operative in *M. tuberculosis* during phosphate limiting conditions during infection (Banerjee et al., 2000). The *pst* operon encodes pseudogenes in *M. leprae*. As PstB is non-functional in *M. leprae* and active in *M. tuberculosis* this may indicate that *M. leprae* encounters stable phosphate content within the host, making the presence of PstB unnecessary. Besides its role on phosphate uptake, PstB is also associated to the extrusion of antibiotics (Table 1). Rv1473 and Rv2477c encode putative macrolide transporters with functional orthologues in *M. leprae* genome. To our knowledge, there is no evidence for *M. leprae* isolates resistant to clarithromycin so far, stressing the therapeutic usefulness of this antibiotic against *M. leprae*.

ABC transporters encoded by “two genes” are Rv1218c-Rv1217c, Rv1273c-Rv1272c, Rv1668c-Rv1667c, and Rv1687c-Rv1686c. Although nothing is known about their expression in *M. tuberculosis*, the orthologous genes of *Rv1668c*-*Rv1667c* and *Rv1687c-Rv1686c* in *M. leprae, ML1240c-ML1239c*, and *ML1350c-ML1349c*, respectively, are non-functional. The Rv1218c-Rv1217c efflux pump has been associated with *M. tuberculosis* resistance to a wide variety of chemical classes of compounds (Table 1). Orthologues of Rv1273c-Rv1272c can be found in *M. leprae* genome but because there are no studies about their role on *M. tuberculosis* drug resistance, nothing can be anticipated for *M. leprae*.

ABC transporters encoded by “three genes” are the operons Rv1458c-Rv1457c-Rv1456c, DrrABC and Rv2688c-Rv2687c-Rv2686c associated with the extrusion of several drugs in *M. tuberculosis* (Table 1). *M. leprae* orthologue of Rv1458c-Rv1457c-Rv1456c is ML0590c-ML0589c-ML0587c, which encodes a functional efflux transporter. In relation to Rv2688c-Rv2687c-Rv2686c, *M. leprae* chromosome has non-functional orthologues of two components, *ML1034*-*ML1035* (positive strand), while the orthologue of *Rv2688c* is absent. The genes *Rv2686c, Rv2687c*, and *Rv2688c* are co-transcribed. Rv2686c and Rv2687c proteins possess six transmembrane segments, whereas Rv2688c has a nucleotide-binding domain and is likely involved in ATP hydrolysis. In consequence, Rv2688c probably coordinates the functionality of the whole operon. In this case, it is not surprising that the absence of the Rv2688c orthologue in *M. leprae* renders non-functional the other genes within this operon, contributing to the increased susceptibility of *M. leprae* to fluoroquinolones.

### MFS Transporters

*M. leprae* genome possess 11 MFS drug transporters, whereas 20 can be detected in *M. tuberculosis* (Table 1). Among the nine transporters absent from *M. leprae* genome, nothing is known about their role in *M. tuberculosis* for five of them, while for the remaining four it has been described an association with resistance to several drugs (Table 1). Concerning the 11 MFS efflux transporters present in *M. leprae*, eight are non-functional of which some were found to be upregulated in *M. tuberculosis* in response to antibiotics (Table 1). Among these is noted that Rv1258c, also known as Tap-like efflux pump, is a pseudogene (*ML1104c*) in *M. leprae* presenting 58.4% similarity at nucleotide level with *Rv1258c*. Rv1258c is associated with reduced susceptibility to several drugs, namely to rifampicin and clofazimine (Table 1) and has an essential role in physiology, growth, and cell morphology (Ramón-García et al., 2012). These findings emphasize the important role of the Rv1258c efflux pump in the oxidative stress response, cell wall assembly and growth, intrinsic drug resistance (Ramón-García et al., 2012) and macrophage tolerance (Adams et al., 2011). Since *M. leprae* orthologue is non-functional, it is unlikely that Rv1258c play any role in *M. leprae* intrinsic drug resistance and virulence. This can be one more genomic trait of *M. leprae* that contributes to its notable susceptibility to rifampicin and clofazimine *in vivo*.

*M. leprae* chromosome encodes only three functional MFS efflux pumps associated with drug transport in *M. tuberculosis* (Table 1). Of these, the *M. tuberculosis* P55 efflux pump (Rv1410c), orthologue of *M. leprae* ML0556c, is one of the most relevant and well-studied efflux pumps of *M. tuberculosis* and has been associated with the resistance to several drugs (Table 1) and virulence. P55 forms an operon with LprG (Rv1411c), a conserved lipoprotein, which is required for *in vivo* growth of *M. tuberculosis* (Bigi et al., 2004; Farrow and Rubin, 2008), virulence (Bianco et al., 2011a) and accurate cell-wall assembly (Bigi et al., 2004; Bianco et al., 2011b). *M. leprae* encodes both proteins presenting high similarity with those of *M. tuberculosis*. P55 is also associated with cholesterol transport, carbon metabolism, and oxidative stress, which are of major importance for mycobacterial optimal survival and pathogenesis (Ramón-García et al., 2015). Contrary to that observed for Rv1258c (Tap-like efflux pump), the presence of P55 in *M. leprae* genome indicates a vital role of this transporter in *M. leprae* for which a significant contribution in providing intrinsic antibiotic resistance is plausible.

### MATE Transporters

DinF (Rv2836c) is the only MATE transporter that *M. tuberculosis* genome encodes. The *M. tuberculosis* homologue in *M. smegmatis* (Mmp) is involved in the resistance to multiple drugs (Table 1). Importantly, DinF is absent from *M. leprae* genome which can be related with the Na^+^-dependent nature of the MATE transporters that may not exist in the environment where *M. leprae* resides. Youm and Saier (2012) also noted the absence of other NA^+^ transporters in *M. leprae* that are present in *M. tuberculosis* and suggested that these facilitators probably contribute to the maintenance of ion homeostasis and adaptation to several stress conditions.

### SMR Transporters

*M. tuberculosis* genome harbors only one gene belonging to the SMR family, the *mmr* gene, orthologue of *ML1756* of *M. leprae*. Mmr overexpression was showed to decrease susceptibility of *M. smegmatis* and *M. tuberculosis* to intercalating dyes, quaternary ammonium compounds and antibiotics (Table 1). Mmr is controlled by the TetR-like transcriptional repressor Rv3066 (Bolla et al., 2012) whose orthologue in *M. leprae* is ML1757. In both species, the transcriptional repressor is located immediately downstream of *mmr* or *ML1756*. However, while in *M. tuberculosis* the Rv3066 gene encodes a 202-aminoacidic protein, its orthologue in *M. leprae* is a pseudogene. This means that *mmr* transcription is no longer repressed in *M. leprae*. Nothing is known about *M. leprae* susceptibility to biocides and dyes thus the significance of *mmr* depression cannot be unveiled.

### RND Transporters

*M. tuberculosis* genome contains 13 genes that encode MmpLs (Mycobacterial membrane protein, Large), and five auxiliary proteins, the MmpSs (Mycobacterial membrane protein, Small) (Table 1). The MmpLs efflux pumps are responsible for the transport of lipids, mainly mycolic acids, essential for mycobacterial survival and pathogenesis, and heme transport (Cox et al., 1999; Camacho et al., 2001; Converse et al., 2003; Domenech et al., 2005; Tullius et al., 2011; Grzegorzewicz et al., 2012; Tahlan et al., 2012; Rodríguez et al., 2013). The expression of *M. tuberculosis* MmpL proteins is controlled by a complex regulatory network that includes orthologues of TetR (Rv1816 and Rv3249c) and MarR (Rv0678) transcriptional regulators (Radhakrishnan et al., 2014; Delmar et al., 2015). The transcriptional regulator Rv0678 has no orthologue in *M. leprae* and Rv3249c and Rv1816, whose *M. leprae* counterparts are ML0770 and ML0933, are non-functional. This indicate that some of the *M. leprae mmpL* genes are out of regulation and are being constitutively expressed or MmpLs regulation in *M. leprae* involves a different regulatory network from that found in *M. tuberculosis*. Of the 13 MmpLs encoded in *M. tuberculosis*, only MmpL3, MmpL4, MmpL7, MmpL10, and MmpL11 are functional proteins in *M. leprae*, while the remaining are absent or non-functional (Table 1). Of these, only MmpL3 is essential for *M. tuberculosis* survival (Domenech et al., 2005). Moreover, MmpL3 has emerged as a novel therapeutic target in *M. tuberculosis* (Li et al., 2014). Due to high degree of similarity between *M. leprae* MmpL7, MmpL11, and MmpL3 and their orthologous in *M. tuberculosis* (Table 1), we can anticipate a similar function for both proteins in *M. leprae*.

MmpS5-MmpL5, one of the most important RND transporters of *M. tuberculosis*, is absent in the *M. leprae* genome. During the reductive evolutionary process that *M. leprae* experienced, the MmpS5-MmpL5 efflux transporter was eliminated probably to maintain only the pathways required for a strict intracellular lifestyle, typical of *M. leprae*. The overexpression of the MmpS5-MmpL5 efflux transporter was shown to be associated with resistance of *M. tuberculosis* to azoles (Milano et al., 2009) and bedaquiline and cross-resistance to clofazimine (Andries et al., 2014). The absence of the MmpS5-MmpL5 explains way clofazimine is so efficient against *M. leprae*. So far, very rare *M. leprae* strains were described with clofazimine resistance reinforcing the connection between MmpS5-MmpL5 and clofazimine resistance as well as its unique hypersusceptibility in *M. leprae*.

## Conclusions and Future Prespectives

The occurrence of shared multidrug resistance efflux transporters between *M. leprae* and *M. tuberculosis* reveals implications for drug resistance and virulence. Multidrug resistance efflux pumps are ubiquitous in nature. Some efflux pumps exhibit a dual role in *M. tuberculosis* contributing to both drug resistance and virulence. Here, we have shown that the major *M. tuberculosis* efflux pumps that are associated with drug efflux and virulence have been retained during the reductive evolutionary process that *M. leprae* underwent. These efflux pumps are not only important for substrate transport across the inner membrane but are also responsible for drug resistance by extruding drugs from the periplasm to the outside of the cell. They may confer a selective advantage in hostile environments, therefore contributing to *M. leprae* pathogenicity and acquired drug resistance to therapy as seen in *M. tubreculosis*. It has been recently shown that resistance to effective multidrug therapy, especially in the high burden countries such as Brazil and India, is on rise, with noteworthy rates of resistance especially against rifampicin and dapsone (Cambau et al., 2018). Rifampicin resistance was found in new cases of leprosy that may relate to individual abuse of this antibiotic usage for treating other bacterial infections as it was also seen with ofloxacin resistance although an antibiotic not used for the first-line treatment of leprosy (Cambau et al., 2018). Future work should focus on efflux pumps, as those mentioned above, as new drug targets for new leprosy therapeutics. A comparative transcriptomic profile of these transporters may provide additional insights, since differences are expected in the efflux pump expression due to pathogen specificity as consequence of the obligate intracellular lifecycle of *M. leprae*. The modulation of these novel targets will enhance the eradication efforts of endemic leprosy and prevent emergence of drug resistance in afflicted countries. This comparative and perspective study identified these new targets using biological information gathered from *M. tuberculosis* and constitutes the first step for a more detailed computational studies to bring more mechanistic insights and biological analyses to be applied to *M. leprae*, susceptible and drug resistant clinical strains, similar to what have been done for *M. tuberculosis* (Sandhu and Akhter, 2016). The increase in the number of available sequenced genomes and structural data of these proteins together with the advances on experimental and computational biology will improve our knowledge on the relationship between *M. leprae* protein sequence, structure, dynamics and function (Li et al., 2017).

## Author Contributions

DM and MV designed this conceptual study. DM performed the analyses. DM, EL, FM, EC and MV have made substantial contributions to the work and approved its final version.

### Conflict of Interest Statement

The authors declare that the research was conducted in the absence of any commercial or financial relationships that could be construed as a potential conflict of interest.
